# Neighbourhood, Route and Workplace-Related Environmental Characteristics Predict Adults' Mode of Travel to Work

**DOI:** 10.1371/journal.pone.0067575

**Published:** 2013-06-19

**Authors:** Alice M. Dalton, Andrew P. Jones, Jenna R. Panter, David Ogilvie

**Affiliations:** 1 School of Environmental Sciences, University of East Anglia, Norwich, United Kingdom; 2 Norwich Medical School, University of East Anglia, Norwich, United Kingdom; 3 UKCRC Centre for Diet and Activity Research (CEDAR), Institute of Public Health, Cambridge, United Kingdom; 4 Medical Research Council Epidemiology Unit, Institute of Public Health, Cambridge, United Kingdom; Old Dominion University, United States of America

## Abstract

**Objective:**

Commuting provides opportunities for regular physical activity which can reduce the risk of chronic disease. Commuters' mode of travel may be shaped by their environment, but understanding of which specific environmental characteristics are most important and might form targets for intervention is limited. This study investigated associations between mode choice and a range of objectively assessed environmental characteristics.

**Methods:**

Participants in the Commuting and Health in Cambridge study reported where they lived and worked, their usual mode of travel to work and a variety of socio-demographic characteristics. Using geographic information system (GIS) software, 30 exposure variables were produced capturing characteristics of areas around participants' homes and workplaces and their shortest modelled routes to work. Associations between usual mode of travel to work and personal and environmental characteristics were investigated using multinomial logistic regression.

**Results:**

Of the 1124 respondents, 50% reported cycling or walking as their usual mode of travel to work. In adjusted analyses, home-work distance was strongly associated with mode choice, particularly for walking. Lower odds of walking or cycling rather than driving were associated with a less frequent bus service (highest versus lowest tertile: walking OR 0.61 [95% CI 0.20–1.85]; cycling OR 0.43 [95% CI 0.23–0.83]), low street connectivity (OR 0.22, [0.07–0.67]; OR 0.48 [0.26–0.90]) and free car parking at work (OR 0.24 [0.10–0.59]; OR 0.55 [0.32–0.95]). Participants were less likely to cycle if they had access to fewer destinations (leisure facilities, shops and schools) close to work (OR 0.36 [0.21–0.62]) and a railway station further from home (OR 0.53 [0.30–0.93]). Covariates strongly predicted travel mode (pseudo r-squared 0.74).

**Conclusions:**

Potentially modifiable environmental characteristics, including workplace car parking, street connectivity and access to public transport, are associated with travel mode choice, and could be addressed as part of transport policy and infrastructural interventions to promote active commuting.

## Introduction

People who engage in physical activity are less likely to suffer from a range of preventable chronic diseases [1]. However, most people do not meet government guidelines for physical activity [2], which contributes to the burden of disease in the UK [3] and globally [4]. One way of increasing population activity levels may be to build it into daily routines, for example in the form of active travel (cycling and walking) for part or all of the journey to work [5]. This has the potential to make a significant contribution to physical activity, as commuting to and from work constitutes 15% of the journeys made in the UK and the United States [6], [7] and 19% of the distance travelled [7]. There has been a steady decline in cycling and walking to work during the last few decades as the car has become a more popular choice [8] and people have tended to live further from work [9]. Nevertheless, there is evidence that regular active travellers are physically fitter and have higher levels of mental wellbeing and lower sickness absence from work [10], [11]. In addition, public transport may provide an alternative to the car for those living further from work and offer health as well as environmental benefits, with US research finding that people walking to transit stops can accrue around 30% of their recommended daily activity levels by doing so [12]. A recent modelling study has suggested that a step-change in the prevalence of walking and cycling in England and Wales could save the National Health Service approximately £17bn in healthcare costs associated with chronic conditions such as type-2 diabetes, cancer, heart disease and depression over the next 20 years [13].

Features of the built and natural environments within which people live and work may influence the mode of travel used [1], such as for journeys to work, by either supporting or acting as a barrier against walking and cycling. In order to develop environments more supportive of a modal shift towards more active travel at the population level, we therefore need to better understand the importance of characteristics of the home, work and journey environments [14], [15]. Whilst there is good evidence that modal choice is associated with travel distance, evidence is more equivocal regarding the importance of many other characteristics of the physical environment including street network connectivity, urban design, walkable destinations (including schools, shops, leisure and cultural facilities), land use, infrastructure for walking or cycling, and the availability of or access to public transport [1], [16], [17]. It is also noteworthy that despite the constraints of distance, some people do use active modes of travel even if they live a relatively long way from work.

In part, our limited understanding of the correlates of active travel may reflect methodological limitations of some previous studies. A recent systematic review of the environmental correlates of cycling noted that few studies had measured the environment objectively and none of the 21 studies identified could be regarded as methodologically strong [16]. Reasons included failing to control for confounders, such as gender and age; failing to carry out analysis at the individual level; and using inappropriate statistical methods. Many studies have examined environmental attributes, public transport (transit) accessibility, or restrictions on car use singly but not in combination, meaning that the relative importance of these factors is not known. For these reasons, a recent review concluded that empirical research either does not include or provides inconclusive evidence on the influence of the transport environment on travel behaviour [1]. In addition, research has tended to treat walking and cycling as a singular behaviour, whereas they are distinct behaviours likely to have different correlates [18], [19]. A further limitation is that characteristics of the home neighbourhood have often been the focus for analysis, yet the workplace environment and that of the route between home and work may also be important [20], [21], [22]. Although some research has considered distances to public transport (transit) stops [23], overall accessibility and the ease by which that transport may be used to reach work has rarely been considered [16], [24]. In general, previous studies on active travel suggest that existing models fail to account for a significant proportion of the variance in travel behaviour. Environmental attributes have been shown to explain some of this variance, but prior empirical research has typically tested a limited range of independent variables in rather environmentally homogeneous, often urban, settings.

Using a much wider range of objective environmental measures than have commonly been tested, the aim of this study is to investigate the extent to which features of the environment are associated with modal choice amongst a sample of commuters in Cambridge, UK. We focus on the individual level and assess correlates for travel by all modes. In doing so, we aim to identify modifiable environmental characteristics that might form the targets of future interventions to increase the prevalence of active travel.

## Methods

### Study design and setting

This research analysed cross-sectional data obtained from a sample of commuters taking part in the Commuting and Health in Cambridge study in Cambridge, UK. The details of this study have been outlined previously [25]. Participants were aged 16 and over, working in Cambridge and living within 30 km of the city. They were sampled using a workplace recruitment strategy that targeted a variety of workplaces and employers in a range of geographical locations across the city centre and urban fringe.

The data used for this analysis were collected between May and November 2009 using postal questionnaires [26]. Participants reported their recent physical activity (at home, at work and for recreation), general health, and travel to and from work and for other purposes. Personal characteristics such as age, gender, weight, height and highest educational attainment were also reported. 1168 respondents returned questionnaires, 1155 of whom provided valid postcodes which could be used to identify their home and work locations.

### Ethics statement

Ethical approval for the study was obtained from the Hertfordshire Research Ethics Committee (reference number 08/H0311/208) and written informed consent was provided by each participant. No minors/children participated in this study.

### Determining modal choice

Two sections of the survey questionnaire were relevant to this analysis. The first comprised the Recent Physical Activity Questionnaire (RPAQ), a validated instrument that measures physical activity in the previous four weeks [27]. RPAQ was used to classify participants according to their usual mode of travel to work. The survey listed four modes (car/motor vehicle, works or public transport, bicycle, or walking) and participants were asked to specify if they ‘always’, ‘usually’, ‘occasionally’ or ‘never or rarely’ travelled to work by each mode. Most participants selected the option of ‘always’ for one mode of transport, and their usual mode was classified accordingly. For the 178 cases in which more than one mode was identified, rules were established to classify participants according to their predominant modal choice based on the most frequently reported travel mode: if participants stated that they always or usually walked and/or cycled but that they also always or usually used the car, they were coded as using the car as it was presumed this would constitute the main component of the journey (n = 92); if they stated they always or usually walked and/or cycled but also always or usually used public transport, they were coded as using public transport (n = 39); if they reported always or usually walking and cycling they were coded as cycling (n = 9); and if they stated that they sometimes used the car and sometimes the bicycle, they were coded as using the car (n = 3).

For 35 participants, no predominant mode could be determined at this stage. In these cases, a question from the second relevant section of the questionnaire, ‘*About your travel to and from work in the last seven days*’, was used to identify the predominant mode or modes used in the previous seven days. Of those always reporting more than one mode per journey, 19 using the car and bus were classified as car users, one reporting walking and using the train was classified as using the train, and one using the train and the car was classified as using the train. For those reporting a mixture of different modes which varied throughout the week, six used the car most frequently and one used the train most frequently. One participant did not answer the RPAQ question but responded as only using the car to the ‘last seven days’ question. Six participants could not be attributed to one predominant mode because they reported using a mixture of different modes of transport throughout the week with equal frequency, and were therefore not included in subsequent analyses.

### Environmental measures, neighbourhood delineation and route identification

For each participant, environmental measures were calculated for the home neighbourhood, the work neighbourhood, and the route between home and work. Table 1 presents a total of 30 different measures together with the data sources that were used to calculate them, grouped into themes of ‘roads and routes’, ‘public transport’ and ‘land use’.

**Table 1:** Prevalence of travel mode by levels of environmental exposure variables.

			% prevalence				
Variable	Data source	Classification	Car	PT	Bike	Walk	p^1^
***Roads and routes***							
Distance to work (km)	A	Shortest	4.8	3.5	72.8	18.8	<0.001
		Middle tertile	36.6	8.8	53.6	1.1	
		Longest	76.5	19.7	3.7	0.0	
Route directness to work (ratio)	A	Most direct	52.8	19.0	24.7	3.5	<0.001
		Middle tertile	42.8	8.8	44.1	4.3	
		Least direct	22.7	4.3	61.1	12.0	
Direction of travel (category)	B	Lives and works in city	5.9	2.9	76.6	14.7	<0.001
		Lives in city, works outside	15.1	7.0	66.6	11.4	
		Lives outside, works in city	58.8	26.1	15.1	0.0	
		Lives and works outside city	77.5	8.8	13.7	0.0	
Proportion of A & B roads (%, route)	A	Lowest	28.7	7.8	52.3	11.3	<0.001
		Middle tertile	31.0	7.2	56.2	5.6	
		Highest	58.6	17.1	21.4	2.9	
Road density (ratio, home)	A	Highest	27.1	12.8	50.3	9.8	<0.001
		Middle tertile	35.6	11.8	46.5	6.1	
		Lowest	55.6	7.5	33.2	3.7	
Junction density (ratio, home)	A	Highest	26.6	15.4	48.1	9.8	<0.001
		Middle tertile	34.0	10.2	48.8	7.0	
		Lowest	57.6	6.4	33.2	2.9	
Road connectivity (ratio, home)	A	Most connected	22.9	8.1	55.8	13.2	<0.001
		Middle tertile	42.8	11.2	41.5	4.5	
		Least connected	52.3	12.7	32.9	2.1	
Existence of A roads (category, home)	A	None^2^	53.3	13.1	30.2	3.4	<0.001
		Some	29.3	8.9	52.8	8.9	
Proportion foot/cycle paths (%, home)	A	Highest	52.2	10.8	32.3	4.8	<0.001
		Middle tertile	36.4	14.0	44.9	4.7	
		Lowest	29.8	7.2	52.8	10.2	
Effective walkable area (ratio, home)	A	Most walkable	24.8	12.5	49.9	12.8	<0.001
		Middle tertile	40.8	10.1	45.6	3.5	
		Least walkable	52.7	9.4	34.5	3.5	
***Public transport***							
Bus service to work (category)	C, D	Direct	30.6	9.5	51.1	8.8	<0.001
		Change	65.0	14.6	20.1	0.4	
		None	46.9	6.3	43.8	3.1	
Distance to nearest bus stop (m, home)	C	Closest	35.1	13.0	45.5	6.4	0.043
		Middle tertile	41.2	12.0	39.3	7.5	
		Furthest	42.0	7.0	45.2	5.9	
Distance to nearest bus stop (m, work)	C	Closest	44.8	11.5	36.3	7.5	0.001
		Middle tertile	31.8	12.5	48.2	7.5	
		Furthest	41.1	7.9	46.3	4.7	
Bus service frequency (count, home)	C, D	Most frequent	11.1	7.4	68.3	12.9	<0.001
		Middle tertile	41.6	11.7	41.9	4.8	
		Least frequent	62.9	12.6	21.8	2.6	
Bus service frequency (count, work)	C, D	Highest	35.3	11.2	47.6	5.9	0.005
		Middle tertile	35.2	14.2	44.4	6.2	
		Lowest	45.4	7.8	39.3	7.4	
Nearest railway station (m, home)	C	Closest	24.3	14.1	50.1	11.5	<0.001
		Middle tertile	23.7	6.7	61.5	8.1	
		Furthest	69.8	11.1	18.8	0.3	
Nearest railway station (m, work)	C	Closest	26.8	13.4	50.1	9.7	<0.001
		Middle tertile	45.0	9.3	39.2	6.4	
		Furthest	46.7	9.3	41.0	3.0	
Number of bus stops (count, home)	C	Highest	20.1	10.2	58.4	11.3	<0.001
		Middle tertile	40.4	13.0	42.5	4.2	
		Lowest	60.7	9.2	26.8	3.3	
Number of bus stops (count, work)	C	Upper half^2^	34.5	12.5	45.7	7.3	0.006
		Lower half	44.2	8.9	41.0	5.9	
Number of bus routes (count, home)	C	Highest	23.4	10.4	53.8	12.5	<0.001
		Middle tertile	41.1	12.7	42.3	3.9	
		Lowest	57.5	8.0	31.4	3.0	
Number of bus routes (count, work)	C	Upper half^2^	31.0	12.2	48.8	8.0	<0.001
		Lower half	46.4	9.4	38.8	5.4	
***Land use***							
Land use mix (score, route)	E, F, G	Most mixed	45.6	11.5	39.5	3.5	<0.001
		Middle tertile	28.5	9.3	54.9	7.2	
		Least mixed	44.1	11.2	35.6	9.1	
Land use mix (score, route)	E, F, G	Most mixed	39.1	11.8	42.1	7.0	0.197
		Middle tertile	38.0	13.2	42.5	6.3	
		Least mixed	41.1	7.0	45.4	6.5	
Building density (%, route)	G	Highest	8.6	4.6	73.2	13.7	<0.001
		Middle tertile	40.3	9.8	43.8	6.1	
		Lowest	69.3	17.6	13.1	0.0	
Building density (%, home)	G	Highest	16.9	10.7	59.8	12.6	<0.001
		Middle tertile	42.6	12.0	39.6	5.9	
		Lowest	58.7	9.3	30.7	1.3	
Number of destinations (count, route)	H	Highest	20.7	6.7	61.8	10.8	<0.001
		Middle tertile	37.3	14.4	42.0	6.3	
		Lowest	60.4	10.8	26.1	2.7	
Number of destinations (count, home)	H	Highest	17.1	10.5	59.6	12.9	<0.001
		Middle tertile	42.0	9.8	43.4	4.8	
		Lowest	59.9	11.7	26.4	1.9	
Number of destinations (count, work)	H	Upper half^2^	31.3	13.3	47.5	8.0	<0.001
		Lower half	47.2	8.2	39.4	5.2	
Deprivation (%, home)	I	Lowest	26.3	4.8	57.4	11.5	<0.001
		Middle tertile	42.9	11.2	40.5	5.3	
		Highest	48.9	16.0	32.2	2.9	
Car parking availability (category, work)	J	Yes - pay to park	45.4	9.7	40.0	4.9	<0.001
		Yes - free	47.6	8.2	39.9	4.3	
		No	24.2	14.4	50.6	10.8	

Notes: n = 1124. PT: public transport.

1 Pearson chi-square.

2 Data divided into two categories using median value (none/some, upper/lower half) when tertiles produced uneven numbers due to multiple participants working in the same location. *Data sources*: A Ordnance Survey (OS) road centre lines [50], Cambridge County Council, Sustrans [30], OpenStreetMap [51] (various dates) and manually digitised; B questionnaire 2009 [26]; C DfT 2010 [35]; D DfT 2009 [34]; E CeH 2000 [52]; F OS 2010 [53]; G Natural England [54], OS [53] and OpenStreetMap [51] (various dates); H PointX Ltd 2010 [55]; I ONS 2001 (proportion of people in semi-routine occupations, routine occupations, never worked and long-term unemployed categories) [56]; J questionnaire 2009 [26].

In order to generate neighbourhoods, participants' home and work postcodes were georeferenced using the Ordnance Survey (OS) Address Layer 2®database [28]. A pedestrian route network dataset was constructed in the ArcGIS 9.3 [29] geographic information system (GIS) software package by combining road data, excluding motorways, from the OS MasterMap® Integrated Transport Network™ (ITN) database with local authority rights-of-way data (public footpaths, bridleways and byways), cycle route information from the charity Sustrans [30], and other informal pathways recorded on OpenStreetMap.com. Using this information, home neighbourhoods were delineated to represent areas that could be accessed within an approximate ten-minute walking time (equating to 800 m along the pedestrian network). This distance has been used in previous accessibility analysis to represent a practically walkable neighbourhood area [19], [31], [32].

To delineate the route between home and work for each participant, ArcGIS was used to identify sections of the pedestrian network that comprised the shortest route between the two locations. Subsequently, the characteristics of the environment within a 100 m distance of the route were quantified.

The ‘roads and routes’ environmental variables that were computed included a measure of distance to work (length of shortest route between home and work), route directness (ratio of route network to straight line distance), whether participants were travelling into or out of the city centre on their journey from home to work (a participant was defined as working in the city if they worked within 2 km of Cambridge central bus station, and defined as living in the city if they lived in the Cambridge urban area according to the 2001 census [33]) and the proportion of route length that was along A or B (major) roads. In addition, five measures of the walkability of the streets in the home neighbourhood were calculated, including road density, junction density, road connectivity, existence of A class (major) roads, the proportion of foot/cycle paths, and the general ‘walkable area’ (defined as the area walkable within 800 m along the route network buffer from participants' home location divided by the area within an 800 m straight line distance).

Public transport variables were derived using the route network dataset in combination with the National Public Transport Access Nodes (NAPTAN) and Data Repository (NPTDR) datasets [34], [35]. Firstly, it was established whether or not there was a bus service passing through the neighbourhood that would take the participant to work, either directly or with one or more changes. The other variables calculated for both the home and work locations were the distance to the nearest bus stop, bus service frequency, the number of bus stops, the number of bus routes served and the distance to the nearest railway station. These indicators used the pedestrian route network, the frequency of bus services from the nearest bus stop on a typical weekday, the number of serviced bus stops present, and the number of bus routes available in the neighbourhood based on a count of unique service numbers.

Land use variables included an indicator of land use mix - the Herfindahl-Hirschmann Index (HHI) [36] - and building density at the home location and on the route to work. A count of the number of destinations (schools, eating and drinking establishments, stage and screen venues, sports complexes, and retail units) was taken after they were mapped along the route to work and in the home and work neighbourhoods. A measure of local area deprivation was calculated for the home neighbourhood, based on the proportion of people in lower socioeconomic classes (semi-routine occupations, routine occupations, never-worked and long-term unemployed categories) living within the relevant Census Output Area. In addition, the availability of workplace car parking (none, charged or free) reported by each participant in their questionnaire was used.

### Data analysis

Unadjusted associations between usual travel mode (car, public transport, cycling or walking) and home, work, and route characteristics were examined using chi-square tests and analyses of variance. Continuous variables were categorised as tertiles or using other appropriate groupings. Prior to model fitting, multicollinearity was managed using a pair-wise correlation matrix to identify variables that were highly associated, defined as having a Pearson's correlation coefficient of >0.55 based on previous empirical research [37]. Only one variable from each correlated pair was added into each model, the chosen one being that with the strongest association in the expected direction with the outcome variable. Multinomial regression models were then fitted to examine adjusted associations, using commuting by car as the reference category for the outcome variable. The models were fitted in a number of stages. First, a personal model was created by entering all potential individual and household-level covariates into a multinomial logistic regression. Prior empirical evidence has shown mixed associations between individual factors and active travel [17], therefore all such variables were included at this stage. After all these potential predictors were added, those for which p>0.1 were removed in a backwards stepwise manner leaving only those which were statistically significant at p<0.1.

Next, in order to establish the potential environmental correlates, three further models were fitted using the same method for the measures grouped into the ‘roads and routes’, ‘public transport’ and ‘land use’ categories. The variables that remained statistically significant at p<0.1 in each of the three models were then combined with those from the individual model, and non-significant independent variables were dropped in a stepwise manner using a threshold of p>0.05. The resultant model contained those variables that remained statistically significant at p<0.05. All analyses were undertaken using PASW Statistics 18 [38].

## Results

### Sample characteristics

Of the 1168 respondents who completed the questionnaire, 13 were excluded as postcode data for home location, work location or both were missing or invalid. Of the remaining 1155, six could not be attributed to one predominant mode for commuting, while two worked at more than one site and therefore had no ‘usual’ place of work. Of the remaining sample, 23 participants had missing values for one or more of the covariates adjusted for and were also excluded, resulting in a final sample of 1124 for analysis. There were no statistically significant differences between those included and excluded from analysis in terms of age, gender, limiting illness, body mass index (BMI), type of work, education, home ownership or having children in the household.

The mean age of the sample was 42.3 (range 17.6 to 71). 31.7% were male, whilst 60.5% were of normal weight, 27.6% overweight and 9.4% obese. 80.2% were engaged in sedentary work as opposed to standing (16.3%) or manual (3.6%) work, and 59.4% reported excellent or very good health. 10.0% reported a limiting illness, 72.0% were educated to degree level, 72.4% were homeowners, 29.6% had children under the age of 16 living in the household and 85.7% owned at least one car. Based on the classification by Bibby and Shepherd [39], 65.9% of participants lived in urban areas, 19.4% in town and fringe locations, 12.5% in villages and 2.1% in hamlets and isolated dwellings. In terms of predominant mode of travel to work, 39.4% used the car, 10.7% public transport and 43.3% the bicycle, while 6.6% walked. Therefore, nearly half of respondents (n = 561) predominantly used an active mode of travel for commuting.

### Unadjusted prevalences of travel modes by levels of environmental exposure variables

Table 1 shows the unadjusted percentage prevalence of travel modes according to the three domains of environmental variables. There were statistically significant differences in travel mode prevalence across the categories of all these exposure variables, with the exception of land use mix in home neighbourhoods. Walking was most prevalent in those individuals whose modelled routes to work were less direct, involved the lowest proportion of A or B roads, had many destinations along them, had a direct bus service, passed through areas of high building density and low land use mix, and started and ended within the city. In terms of home neighbourhoods, walking prevalence was highest in areas which were walkable and highly connected, had frequent bus services and a larger number of bus stops and bus routes, had a railway station nearby, a high building, road and junction density, some A roads and few official foot/cycle paths, had low levels of deprivation, and contained many amenities/destinations. For the work location, a higher prevalence of walking was associated with proximity to a railway station, with access to a higher number of bus stops and amenities/destinations, but fewer bus services, within the work neighbourhood, and with not having access to free workplace car parking. Patterns in the percentage prevalence of cycling were similar to those for walking except in the case of bus service frequency at the work location. The prevalence of cycling was highest in areas of high bus service frequency, opposite to the direction of association for walking.

### Multinomial modelling

The multivariable personal model contained nine explanatory variables, with a pseudo r-squared value of 0.40 (Table 2). The three further regression models including the grouped objective environmental criteria had pseudo r-squared values of: roads and routes 0.63; public transport 0.39; land use 0.41.

**Table 2:** Univariate associations between personal and environmental characteristics and main mode of travel to work (reference category is ‘car’).

		Odds Ratio (95% CI)		
		Public transport	Bike	Walk
***Personal characteristics*** * (pseudo r^2^ = 0.400)*				
Age		1.001 (0.980, 1.023)	0.996 (0.981, 1.011)	1.014 (0.987, 1.042)
Gender	Female (ref)	1.00	1.00	1.00
	Male	1.301 (0.777, 2.179)	2.450 (1.734, 3.462)***	1.591 (0.870, 2.910)
Limiting illness	No (ref)	1.00	1.00	1.00
	Yes	0.465 (0.223, 0.972)**	0.369 (0.218, 0.627)***	0.605 (0.245, 1.492)
Deprivation	Least deprived (ref)	1.00	1.00	1.00
	Middle tertile	1.748 (0.916, 3.338)*	0.499 (0.340, 0.733)***	0.410 (0.214, 0.787)***
	Most deprived	1.751 (0.936, 3.274)*	0.335 (0.226, 0.497)***	0.166 (0.077, 0.356)***
Education	Degree (ref)	1.00	1.00	1.00
	No degree	1.210 (0.761, 1.926)	0.544 (0.381, 0.777)***	0.438 (0.217, 0.884)**
Homeownership	Homeowner (ref)	1.00	1.00	1.00
	Not a homeowner	1.695 (0.975, 2.948)*	1.582 (1.038, 2.412)**	2.559 (1.261, 5.195)***
Children in household	Yes (ref)	1.00	1.00	1.00
	No	0.927 (0.555, 1.550)	0.702 (0.498, 0.989)**	1.339 (0.643, 2.788)
Car ownership	No car (ref)	1.00	1.00	1.00
	1 car	0.058 (0.017, 0.204)***	0.067 (0.020, 0.222)***	0.028 (0.008, 0.101)***
	More than 1 car	0.010 (0.003, 0.038)***	0.012 (0.004, 0.041)***	0.006 (0.001, 0.022)***
Type of work	Sedentary (ref)	1.00	1.00	1.00
	Standing	0.955 (0.521, 1.751)	1.247 (0.822, 1.893)	1.059 (0.499, 2.247)
	Manual	0.760 (0.230, 2.518)	0.988 (0.433, 2.253)	2.809 (0.861, 9.164)*
***Roads and routes characteristics*** * (pseudo r^2^ = 0.630)*				
ROUTE				
Distance to work (km)		0.990 (0.967, 1.014)	0.725 (0.694, 0.757)***	0.277 (0.210, 0.365)***
HOME NEIGHBOURHOOD				
Junction density (ratio)	Highest (ref)	1.00	1.00	1.00
	Middle tertile	0.524 (0.316, 0.867)**	0.572 (0.346, 0.947)**	0.446 (0.210, 0.947)**
	Lowest density	0.217 (0.124, 0.378)***	0.498 (0.294, 0.843)***	0.373 (0.142, 0.978)**
Effective walkable area (ratio)	Most walkable (ref)	1.00	1.00	1.00
	Middle tertile	0.615 (0.367, 1.029)*	0.908 (0.553, 1.490)	0.550 (0.238, 1.272)
	Least walkable	0.520 (0.307, 0.882)**	0.530 (0.321, 0.874)**	0.496 (0.205, 1.200)
***Public transport characteristics*** * (pseudo r^2^ = 0.390)*				
HOME NEIGHBOURHOOD				
Distance to nearest railway station (m)	Closest (ref)	1.00	1.00	1.00
	Middle tertile	0.382 (0.212, 0.687)***	0.762 (0.512, 1.135)	0.408 (0.222, 0.749)***
	Furthest distance	0.304 (0.187, 0.493)***	0.163 (0.110, 0.241)***	‡
Distance to nearest bus stop (m)	Closest (ref)	1.00	1.00	1.00
	Middle tertile	0.792 (0.486, 1.291)	0.783 (0.531, 1.153)	1.086 (0.559, 2.108)
	Furthest distance	0.438 (0.253, 0.761)***	0.763 (0.519, 1.121)	0.782 (0.391, 1.564)
Nearest bus stop service frequency (count)	Highest (ref)	1.00	1.00	1.00
	Middle tertile	0.463 (0.245, 0.874)**	0.256 (0.165, 0.399)***	0.166 (0.083, 0.333)***
	Lowest frequency	0.279 (0.148, 0.526)***	0.080 (0.050, 0.127)***	0.052 (0.023. 0.118)***
WORK NEIGHBOURHOOD				
Distance to nearest railway station (m)	Closest (ref)	1.00	1.00	1.00
	Middle tertile	0.363 (0.221, 0.597)***	0.365 (0.249, 0.535)***	0.293 (0.159, 0.539)***
	Furthest distance	0.382 (0.221, 0.659)***	0.434 (0.289, 0.653)***	0.171 (0.075, 0.390)***
***Land use characteristics*** * (pseudo r^2^ = 0.410)*				
ROUTE				
Building density (%)	Highest (ref)	1.00	1.00	1.00
	Middle tertile	0.611 (0.303, 1.232)	0.140 (0.091, 0.217)***	0.113 (0.060, 0.216)***
	Lowest density	0.626 (0.323, 1.214)	0.024 (0.015, 0.039)***	‡
WORK NEIGHBOURHOOD				
Destinations within walking distance (count)	Highest (ref)	1.00	1.00	1.00
	Lowest	0.475 (0.280, 0.804)***	0.819 (0.559, 1.201)	0.888 (0.452, 1.746)
Car parking availability (category)	No (ref)	1.00	1.00	1.00
	Yes - pay to park	0.627 (0.333, 1.178)	0.633 (0.390, 1.025)*	0.357 (0.157, 0.811)**
	Yes - free parking	0.362 (0.215, 0.609)***	0.729 (0.483, 1.098)	0.411 (0.206, 0.820)**

* Notes: n = 1124. P values reflect difference from reference category *p<0.1,

** **p<0.05;

*** ***p<0.01; ‡ coefficient suppressed where n<3.

The full final multinomial model is shown in Table 3. The odds of cycling were higher in men and those without a limiting illness and lower in those with no degree, with children in the household, and owning one or more cars. The odds of walking were higher in men and those undertaking manual work, and decreased with increasing car ownership. The odds of using public transport also decreased with increasing car ownership. As expected there was a strong decline in walking and a less strong but still statistically significant decline in cycling with increasing route length. Using the reciprocal of the distance-to-work odds ratios in Table 3, the estimated odds of walking or cycling (relative to driving) were 3.9 times and 1.3 times lower, respectively, for each additional kilometre between home and work.

**Table 3:** Adjusted associations (from best-fit multivariable model) between personal and environmental characteristics and main mode of travel to work (reference category is ‘car’) (pseudo r^2^ = 0.738).

		Odds Ratio (95% CI)		
		Public transport	Bike	Walk
***Personal characteristics***				
Age		0.996 (0.974, 1.019)	1.002 (0.983, 1.022)	1.014 (0.982, 1.046)
Gender	Female (ref)	1.00	1.00	1.00
	Male	1.099 (0.603, 2.001)	3.952 (2.403, 6.499)***	2.618 (1.185, 5.783)**
Limiting illness	No (ref)	1.00	1.00	1.00
	Yes	0.516 (0.227, 1.169)	0.295 (0.141, 0.618)***	0.418 (0.125, 1.396)
Deprivation	Least deprived (ref)	1.00	1.00	1.00
	Middle tertile	1.327 (0.638, 2.758)	0.894 (0.533, 1.500)	0.886 (0.383, 2.047)
	Most deprived	1.553 (0.769, 3.137)	0.685 (0.398, 1.179)	0.399 (0.147, 1.082)*
Education	Degree (ref)	1.00	1.00	1.00
	No degree	1.255 (0.756, 2.083)	0.577 (0.359, 0.929)**	0.634 (0.265, 1.515)
Children in household	Yes (ref)	1.00	1.00	1.00
	No	0.723 (0.410, 1.277)	0.548 (0.341, 0.879)**	1.468 (0.595, 3.622)
Car ownership	No car (ref)	1.00	1.00	1.00
	1 car	0.047 (0.012, 0.176)***	0.140 (0.038, 0.509)***	0.056 (0.013, 0.236)***
	More than 1 car	0.011 (0.003, 0.042)***	0.039 (0.010, 0.144)***	0.029 (0.006, 0.136)***
Type of work	Sedentary (ref)	1.00	1.00	1.00
	Standing	1.124 (0.578, 2.187)	1.225 (0.686, 2.187)	1.063 (0.405, 2.792)
	Manual	0.977 (0.271, 3.528)	0.923 (0.301, 2.830)	9.249 (1.768, 48.38)***
***Roads and routes***				
Distance to work (km)		0.996 (0.974, 1.019)*	0.753 (0.716, 0.793)***	0.255 (0.185, 0.352)***
Junction density (ratio, home neighbourhood)	Highest (ref)	1.00	1.00	1.00
	Middle tertile	0.744 (0.412, 1.342)	0.593 (0.335, 1.051)*	0.409 (0.173, 0.967)**
	Lowest density	0.450 (0.235, 0.862)**	0.483 (0.259, 0.901)**	0.217 (0.070, 0.666)***
***Public transport***				
Distance to nearest railway station (m, home)	Closest (ref)	1.00	1.00	1.00
	Middle tertile	0.474 (0.236, 0.949)**	0.504 (0.284, 0.893)**	0.591 (0.259, 1.352)
	Furthest distance	0.382 (0.216, 0.676)***	0.528 (0.298, 0.934)**	‡
Distance to nearest bus stop (m, home)	Closest (ref)	1.00	1.00	1.00
	Middle tertile	0.623 (0.353, 1.101)	0.651 (0.385, 1.101)	0.889 (0.376, 2.102)
	Furthest distance	0.388 (0.209, 0.718)***	0.806 (0.485, 1.342)	1.580 (0.625, 3.992)
Nearest bus stop service frequency (count, home)	Highest (ref)	1.00	1.00	1.00
	Middle tertile	0.450 (0.215, 0.946)**	0.428 (0.241, 0.761)***	0.275 (0.112, 0.674)***
	Lowest frequency	0.322 (0.143, 0.727)***	0.432 (0.226, 0.827)**	0.607 (0.199, 1.847)
***Land use***				
Destinations within walking distance (count, work)	Highest (ref)	1.00	1.00	1.00
	Lowest	0.405 (0.220, 0.748)***	0.364 (0.214, 0.621)***	0.483 (0.201, 1.160)
Car parking availability (category, work)	No (ref)	1.00	1.00	1.00
	Yes - pay to park	0.789 (0.380, 1.640)	1.334 (0.690, 2.578)	0.919 (0.323, 2.613)
	Yes - free parking	0.351 (0.191, 0.647)***	0.548 (0.317, 0.948)**	0.239 (0.097, 0.592)***

Notes: n = 1124. All associations adjusted for all other variables listed in the table. P values reflect difference from reference category *p<0.1,

**p<0.05;

***p<0.01; ‡ findings suppressed where n<3.

Some associations with environmental exposures remained significant after adjustment. Four environmental measures of the home neighbourhood were found to be associated with modal choice. Low junction density (indicating poor street connectivity) and a greater distance to a railway station were associated with lower odds of walking, cycling, and public transport use. A greater distance to the nearest bus stop and a lower bus frequency were associated with lower odds of public transport use, with the odds of cycling also being reduced amongst those living in neighbourhoods with fewer bus services. Only two measures of the work neighbourhood were associated with modal choice. Those working in areas with fewer destinations were less likely to use public transport or cycle, whilst the availability of free parking was associated with lower odds of using public transport, cycling or walking. The Nagelkerke pseudo r-squared value for the final model was 0.74, suggesting that the outcomes were generally well predicted. This value fell to 0.58 when distance to work was removed from the model, highlighting the importance of that characteristic, and to 0.51 when car ownership was also removed. When distance and all other environmental variables were removed from the final model, retaining only the individual characteristics (including car ownership), the value fell to 0.39.

## Discussion

In expanding the range of potential correlates considered, this research sought to address gaps in our understanding of the relative importance of environmental factors that may contribute to explaining the prevalence of active travel, and therefore serve as potential targets for future interventions. Using questionnaire data from the Commuting and Health in Cambridge study, Goodman et al. [40] have previously illustrated how car commuting allows people to accommodate other lifestyle goals, such as home ownership and children, with their daily work commitments. Using the same dataset, Panter et al. [26] have highlighted the importance of distance as a correlate of time spent walking or cycling to work, showing that if people live near enough to work, they may choose to walk or cycle despite having access to a car. As distance is less amenable to intervention than some other environmental characteristics, this study has built on those findings by establishing a range of objective environmental factors other than distance that are associated with modal choice. It has thereby highlighted a number of environmental features that may be amenable to modification and could therefore represent targets for interventions to increase the prevalence of active commuting.

As anticipated, we found travel distance and car ownership to be strong predictors of mode of travel to work. After adjustment we found that public transport provision was associated with its use, whilst neighbourhoods with higher street connectivity (measured by junction density) were associated with higher odds of walking and cycling. The absence of free car parking at work was associated with a markedly higher likelihood of walking, cycling, and public transport use. Many other variables shown to influence modal choice in previous research (e.g. land use mix, the availability, type or directness of roads and paths, or area-level measures of social deprivation) were not found to be significant predictors in the final multivariable model for this study. We do not know the reasons for this, but it may reflect differences in measurement methods employed by different studies, or the fact that we adjusted for a wider range of covariates than is common in the literature. It may also be that the influences on active travel, other than distance, are quite context-specific.

By testing associations with a diverse set of measures based on the objective characterisation of the environment in a well-characterised population cohort, we believe this study has a number of methodological strengths. In particular we examined the potential importance of public transport availability in addition to features of the built environment, thus addressing evidence gaps highlighted by Mackett et al. [22]. We also modelled associations with characteristics of the environment outside the home neighbourhood, which was highlighted as an empirical gap by several papers [20], [21], [22], and the fact we found differences in associations with the home, work and route environments highlights the importance of doing so. This study also analysed walking and cycling behaviour separately rather than combining the two, as recommended by a number of authors [18], [19], and we found some differences in the pattern of associations between them. In addition, the study participants lived in a mixture of urban and rural environments.

There are a number of limitations to this study. Our study cohort sample worked in the city of Cambridge, UK, and lived within a 30 km radius of the centre. It may therefore not be representative of other areas or indeed of the general population of the study area, as illustrated by the fact that 44% of study participants predominantly used the bicycle to travel to work compared with 18.3% of people working in Cambridge according to the 2001 UK Census [41]. The prevalence of cycling in Cambridge is substantially higher than the national average of 3.1%, although a benefit of the high prevalence of cycling in our sample is that it provided us with the statistical power to model this outcome separately from that of walking. Given the high prevalence of cycling in Cambridge, we do not know how readily our results may be generalizable to other locations where cycling culture is less strongly embedded. However, it appears unlikely that the associations we have observed would be completely different in cities that are otherwise similar, and the replicability of our findings could be tested in future studies drawing on samples with greater geographical heterogeneity.

Further limitations include the absence of participants reporting walking as their predominant mode of travel outside the city, which meant that we could not test associations with certain potentially important environmental attributes such as urban-rural status. Almost 40% of our participants worked in one particular campus site, which may have limited the heterogeneity of workplace neighbourhood exposures within the sample, although these participants nonetheless reported varied car parking provision. We were not able to adjust our analyses for income at the individual or household level, for which data were not available, but educational attainment, home ownership and area-level deprivation were all included in the models. The large number of statistical tests we have performed raises the possibility that some of the associations detected could have arisen by chance, and the cross-sectional study design means that it is not possible to determine if the associations are causal. We therefore cannot tell if modifying the characteristics we have found to be associated with our target behaviours would lead to changes in travel behaviour, although the evidence from studies such as this can generate hypotheses to be tested in subsequent intervention studies. A further limitation is that we do not know the motivations for the choice of residential location in our sample, and it may be that certain individuals had migrated to neighbourhoods that were supportive of their preferred travel behaviours. Nevertheless, recent research findings suggest that environmental influences still remain associated with active travel and physical activity even after controlling for this potential self-selection bias [14], [42], [43]. We defined neighbourhood size as 800 m based on an approximate ten-minute walk, which may not necessarily be the most appropriate scale. However there is evidence to suggest that that the choice of neighbourhood size or scale does not strongly affect associations between urban form and travel behaviour [44] or health outcomes [45], even though measures of the environment do vary with scale. Lastly, we used modelled routes to represent the journey to work and these may not necessarily reflect the routes actually taken.

## Conclusions

Because of the constraints of distance, it may be unrealistic to expect a modal shift towards walking and cycling as the predominant modes of travel to work amongst a substantial proportion of the commuting population. However, our findings suggest that the provision of good public transport is associated with higher levels of public transport use, and from a physical activity perspective, this may still be advantageous amongst those living further from work. Our results also suggest that discouraging the provision of free parking in and around the workplace might help to promote walking and cycling to work. In combination with the findings of other recent cross-sectional analyses of environmental factors associated with commuting behaviour [26], [40], [46], [47], our results will help guide the selection of environmental exposure variables for longitudinal analyses of the determinants of behaviour change over time, as well as the design and specification of future intervention studies.

The implications for future transport policy are evident. In response to the growing demand for housing, new urbanised areas – sometimes remote and inadequately linked [48] – are being created where providing frequent public transport connections close to people's homes may not always be economically or politically viable [49]. Therefore, it may be more prudent to encourage people to use existing public transport networks to break their car journeys, for example by encouraging people to ‘park-and-ride’ at public transport intersections or places where they are able to change to active modes of travel for part of their journey. Our results also suggest that future initiatives seeking to modify environmental features that may promote more active travel behaviours should not only focus on residential neighbourhoods but also encompass characteristics of workplace surroundings as well as transport corridors.
